# Enhanced breast cancer diagnosis using modified InceptionNet-V3: a deep learning approach for ultrasound image classification

**DOI:** 10.3389/fphys.2025.1558001

**Published:** 2025-04-22

**Authors:** Samia Allaoua Chelloug, Abduljabbar S. Ba Mahel, Rana Alnashwan, Ahsan Rafiq, Mohammed Saleh Ali Muthanna, Ahmed Aziz

**Affiliations:** ^1^ Department of Information Technology, College of Computer and Information Sciences, Princess Nourah bint Abdulrahman University, Riyadh, Saudi Arabia; ^2^ School of Life Science and Technology, University of Electronic Science and Technology of China, Chengdu, China; ^3^ Institute of Information Technology and Information Security Southern Federal University, Taganrog, Russia; ^4^ Department of International Business Management, Tashkent State University of Economics, Tashkent, Uzbekistan; ^5^ Department of Computer Science, Faculty of Computer and Artificial Intelligence, Benha University, Benha, Egypt; ^6^ Engineering school, Central Asian University, Tashkent, Uzbekistan

**Keywords:** breast cancer, deep learning, InceptionV3, ultrasound images, transfer learning

## Abstract

**Introduction:**

Breast cancer (BC) is a malignant neoplasm that originates in the mammary gland’s cellular structures and remains one of the most prevalent cancers among women, ranking second in cancer-related mortality after lung cancer. Early and accurate diagnosis is crucial due to the heterogeneous nature of breast cancer and its rapid progression. However, manual detection and classification are often time-consuming and prone to errors, necessitating the development of automated and reliable diagnostic approaches.

**Methods:**

Recent advancements in deep learning have significantly improved medical image analysis, demonstrating superior predictive performance in breast cancer detection using ultrasound images. Despite these advancements, training deep learning models from scratch can be computationally expensive and data-intensive. Transfer learning, leveraging pre-trained models on large-scale datasets, offers an effective solution to mitigate these challenges. In this study, we investigate and compare multiple deep-learning models for breast cancer classification using transfer learning. The evaluated architectures include modified InceptionV3, GoogLeNet, ShuffleNet, AlexNet, VGG-16, and SqueezeNet. Additionally, we propose a deep neural network model that integrates features from modified InceptionV3 to further enhance classification performance.

**Results:**

The experimental results demonstrate that the modified InceptionV3 model achieves the highest classification accuracy of 99.10%, with a recall of 98.90%, precision of 99.00%, and an F1-score of 98.80%, outperforming all other evaluated models on the given datasets.

**Discussion:**

The achieved findings underscore the potential of the proposed approach in enhancing diagnostic precision and confirm the superiority of the modified InceptionV3 model in breast cancer classification tasks.

## 1 Introduction

Cancer is a leading cause of death worldwide, making it a critical area of study for improving human health. Characterized by the uncontrolled and disruptive growth of abnormal somatic cells, cancer spreads rapidly and poses significant risks. It is broadly categorized into benign and malignant types. Benign tumors grow relatively slowly and are typically non-malignant, whereas malignant tumors proliferate at an alarming rate and can metastasize, endangering lives (Ara et al., 2021). Among women, breast cancer is one of the most common and deadly forms, alongside brain, lung, bone, blood, and liver cancers. According to the World Health Organization, approximately 2.1 million women are affected by potentially life-threatening breast cancer annually (Solanki et al., 2021). The survival rate is closely tied to tumor size at diagnosis: patients with tumors smaller than 10 mm have a 98% survival likelihood, while 70% of cases are diagnosed when tumors reach 30 mm (Roslidar et al., 2020). Early detection through imaging techniques like X-rays (Din et al., 2022), ultrasound (Allaoua Chelloug et al., 2023), and CT scans (Rafiq et al., 2023) is vital, yet these methods often face limitations, including misclassification of malignant tissues (Loizidou et al., 2023). With an 85% 10-year survival rate in the United States for early-diagnosed cases, and a drop from 98% in stages 0 and I to 65% in stage III (Madej-Czerwonka et al., 2022), the importance of accurate, timely diagnosis cannot be overstated.

Extensive research has been conducted on breast cancer diagnosis, particularly through imaging modalities such as mammography, ultrasonography, and magnetic resonance imaging (MRI). Ultrasonography, which uses high-frequency sound waves to distinguish solid from fluid-filled masses, is often paired with mammography or MRI to enhance diagnostic accuracy (Chahal et al., 2020). However, challenges persist: radiologists misdiagnose approximately 30% of breast malignancies due to the diverse sizes and shapes of masses, and evaluating large volumes of ultrasound images remains difficult even for experts (Chahal et al., 2020). To address this, Computer-aided Diagnosis (CAD) systems have been developed to assist radiologists by analyzing images and highlighting suspicious areas, potentially catching cancers that might otherwise be missed (Elton et al., 2022). Recent advancements in deep learning, particularly convolutional neural networks (CNNs), have improved detection (Elton et al., 2022), classification (Pathak et al., 2020), and segmentation (Byra et al., 2020) of medical images. Despite these advances, limitations remain, including the need for large datasets, which are scarce in medical imaging due to the limited number of patients screened. Transfer Learning (TL) has emerged as a solution, leveraging pre-trained models like AlexNet (Krizhevsky et al., 2012), Inception (He et al., 2016), GoogLeNet (Huang et al., 2017), ShuffleNet (Zhang et al., 2017), and SqueezeNet (Iandola et al., 2016) to overcome data constraints. However, these models, often trained on non-medical datasets like ImageNet (Krizhevsky et al., 2012), struggle with generalization to medical images, leaving room for further improvement in precision and automation for breast ultrasound diagnostics.

This research addresses persistent gaps in breast cancer detection by enhancing the accuracy and efficiency of CAD systems for breast ultrasonography. It focuses on optimizing Transfer Learning to overcome generalization challenges in medical imaging. Specifically, the study refines the Inception V3 model, known for its sophisticated architecture and performance, to develop a less complex yet highly precise diagnostic tool. The work evaluates whether integrating benchmark ultrasound datasets and tailoring pre-trained deep learning models can outperform existing methods, aiming to improve early diagnosis and patient survival rates. Our contributions advance the field of breast cancer diagnostics in several ways.

• We demonstrate that Transfer Learning with pre-trained ImageNet models can achieve exceptional results in detecting breast cancer from ultrasound images.
• We implement data augmentation to enhance model performance and mitigate overfitting, ensuring robustness.
• We evaluate and compare various deep neural network (DNN)-based approaches using metrics such as precision, accuracy, recall, and F-score, providing a comprehensive performance analysis.
• We customize the high-performing Inception V3 model to create an improved CAD system for breast ultrasonography, validated on an integrated dataset of two benchmarking ultrasound image sets. This approach not only boosts accuracy but also enhances generalization, offering a practical tool for radiologists.

The paper is structured as follows: Section 2 reviews related work, Section 3 details materials and methods, Section 4 presents results and discussion, and Section 5 concludes with future directions.

## 2 Related works

Extensive research has been conducted on the application of machine learning (ML) and deep learning (DL) in biomedical imaging, particularly in breast cancer and brain tumor detection. Ragab et al. (2019) proposed a novel CAD approach to classify breast tumors as malignant or benign. Their study utilized two segmentation techniques: one in which the region of interest (ROI) was manually selected and another employing a threshold- and region-based method. The support vector machine (SVM) classifier achieved an area under the curve (AUC) of 94% and an accuracy of 87.2%. To detect breast mass anomalies, Ragab et al. (2013) employed the Discrete Wavelet Transform (DWT) for feature extraction. They compared the performance of SVM and artificial neural networks (ANN) in classifying normal and abnormal tissues, as well as malignant and benign microcalcification (MC) tumors. The ANN and SVM models achieved detection accuracies of 96% and 98%, respectively. Additionally, they integrated deep convolutional neural networks (DCNN) with transfer learning to improve classification performance. When applied to a digital mammographic screening dataset, the proposed approach attained an accuracy of 89.9% in distinguishing between tumor masses and healthy tissues. To further advance breast cancer diagnosis, researchers in (Dubey and Kumar, 2024; Prusty et al., 2023) provide an uncertain expert system for breast cancer prediction, designed to handle the ambiguity and imprecision often present in breast cancer classification. Additionally, researchers in (Hirra et al., 2021) provided a comprehensive review of the latest CAD systems based on deep learning for breast imaging and histopathology. They explored the correlation between histopathological classifications and mammographic findings, considering various biological factors. The study also proposed a computational modeling framework that establishes a relationship between histological representations of mammographic abnormalities and their associated features or phenotypes. The research done by Mondal et al. (2023) introduces an enhanced system architecture for image reconstruction and breast cancer diagnosis with a microwave-tomographic method. Data is acquired by 12 dipole antennas (2.4 GHz), engineered using HFSS software, to simulate the breast structure with differing dielectric characteristics. The data is subsequently processed with the Newton–Kantorovich method to rebuild tomographic images, surpassing alternative techniques such as the gradient method. The program precisely identifies malignant areas, recognizing tissue heterogeneity and providing superior performance relative to leading techniques. The research illustrates the efficacy of microwave imaging in breast cancer detection, offering comprehensive data on tumor dimensions and dielectric characteristics. The simulation outcomes indicate that enhanced algorithms and hardware implementation may augment early-stage breast cancer detection. Khuriwal and Mishra (2018) proposed a Deep Learning Neural Network (DLNN) algorithm for breast cancer detection using the Wisconsin Breast Cancer Database. Their study demonstrated the potential of the UCI dataset in diagnosing breast cancer by implementing a deep learning (DL) approach. While DL techniques are widely applied in fields such as computer vision, image processing, clinical diagnostics, and natural language processing, the authors successfully utilized DL methods to achieve a diagnostic accuracy of 99.67% on the Wisconsin Breast Cancer Database. Their research also compared the proposed DL model with other machine learning algorithms, demonstrating its superior performance. In a related study, Hagos et al. (2018) developed a multi-input CNN designed to incorporate symmetry for breast lump detection. The model was trained on a large dataset comprising 28,294 mammography images. The Area Under the Receiver Operating Characteristic (ROC) Curve (AUC) and the Competition Performance Metric (CPM) were used to evaluate the model’s performance. Without incorporating symmetry, the baseline architecture achieved an AUC of 0.929 with a confidence interval of [0.919, 0.947]. However, when symmetry data was included, the model’s AUC improved to 0.933 with a 95% confidence interval of [0.920, 0.954], highlighting the effectiveness of symmetry-based modeling. Selvathi and Aarthy Poornila (2018) introduced an automated mammogram-based approach for breast cancer detection, utilizing deep learning techniques such as CNNs and stacked sparse autoencoders. Their study evaluated and compared the performance of different algorithms, proposing two frameworks: a single-task CNN and a multi-task CNN incorporating data augmentation and preprocessing. The single-task CNN was employed to diagnose malignancy, whereas the multi-task CNN classified different malignancy levels and image magnifications. The preprocessing steps involved resizing and cropping images to optimize them for CNN input. Their proposed approach achieved a detection accuracy of 83.25%. Additionally, a study (Spanhol et al., 2015) investigated multiclass breast cancer classification using a deep learning model based on DenseNet, a pre-trained convolutional neural network with 201 layers. The classification was conducted on the public BreakHis database, distinguishing both images and patients. The proposed model achieved an image classification accuracy of 95.4% and a patient classification accuracy of 96.48%. Spanhol et al. (2015) further evaluated the algorithm using 600 images from the open-source BreakHis dataset. They utilized the Softmax activation function to compute class probabilities, assigning each test image to the class with the highest probability. As a result, their approach successfully attained an inter-class classification accuracy of 91.5%. The research (Ghosh et al., 2020) thoroughly assesses an automated segmentation technique for breast ultrasound images utilizing DCNNs. The authors provide an innovative CNN architecture that integrates U-Net and a modified ResNet for the automatic segmentation of breast lesions. The suggested method demonstrates substantial enhancements in accuracy, dice coefficient, mean Intersection over Union (IoU), recall, and precision when evaluated on a dataset of 163 pictures, relative to contemporary state-of-the-art techniques. The architecture incorporates a feature extraction unit and utilizes data augmentation approaches to improve training stability and minimize false positives, attaining an accuracy rate of 99%, so establishing it as a potential tool for computer-aided diagnosis in breast cancer detection.

A study by Borah et al. (2022), investigates the application of Vision Transformers (ViT) for the automated diagnosis of breast cancer using mammography pictures. The ViT-based model attained significant accuracy with reduced training duration, rendering it optimal for real-time medical picture interpretation. A graphical user interface (GUI) was created to aid physicians in achieving quicker and more precise diagnosis. The model, validated on the INbreast dataset, attained an accuracy of 96.48%, precision of 93.65%, recall of 93.69%, and an F1-score of 93.34%. The study indicates that ViT is an effective method for breast cancer classification, surpassing current methodologies and demonstrating potential for future applications in medical imaging.

In their study (Sharma and Kumar, 2021), Sharma and Kumar compared handcrafted features with those extracted using the proposed XceptionNet model for breast cancer classification. Their findings demonstrated that the XceptionNet algorithm, employed as a feature extractor, outperformed handcrafted feature extraction techniques. Additionally, the support vector machine (SVM) classifier achieved an accuracy of 96.25% at the ×40 magnification level.

Inan et al. (2022) introduced a comprehensive automated framework for breast tumor segmentation and classification. Their approach relied on segmentation as a preliminary step to enhance classification accuracy. The tumor segmentation was performed using SLIC and K-Means++, while classification was carried out using VGG16, VGG19, DenseNet121, and ResNet50. Among various model combinations, SLIC, U-Net, and VGG16 demonstrated superior performance. Chiu et al. (2020) employed machine learning techniques for breast cancer classification. They utilized principal component analysis (PCA) for feature extraction to address the dimensionality problem and applied a multilayer perceptron (MLP) as the classifier. Similarly, Jabarani et al. (Jebarani et al., 2021) proposed a novel hybrid model for breast cancer detection. Their approach involved specific pre-processing techniques to remove image background noise, employing an adaptive median filter for noise reduction. Additionally, they optimized the K-Means and Gaussian mixture model parameters for image segmentation. The proposed hybrid model achieved an accuracy of 95.5%. Saber et al. (2021) implemented a transfer learning approach with six performance-enhancing matrices in a deep-learning model for automatic breast cancer detection. Feature extraction and classification were conducted using five pre-trained deep learning models: ResNet50, InceptionV3, VGG19, VGG16, and InceptionV2-ResNet. Among these, the VGG16 model demonstrated the highest classification accuracy, achieving 98.96%. Toğaçar et al. (2020) proposed a novel deep learning model for breast cancer classification based on the CNN architecture. The model comprised two primary components: the hyper-column method attention mechanism and a residual block. When applied to the BreakHis dataset, the model achieved an accuracy of 98.80%. Ting et al. (2019) developed an enhanced convolutional neural network architecture for breast cancer classification, termed CNNI-BCC. This supervised deep learning model demonstrated superior performance compared to previous studies, achieving an accuracy of 89.47%. Eroğlu et al. (2021) introduced a hybrid CNN-based model for breast cancer classification. Their approach utilized AlexNet, ResNet50, and MobileNetV2 for feature extraction from ultrasound images of breast cancer patients. The extracted features were optimized using the minimum redundancy maximum relevance (mRMR) technique and subsequently classified using an SVM classifier. The proposed hybrid model achieved a classification accuracy of 95.6%.

Masud et al. (2020) proposed a custom CNN model for breast cancer diagnosis. Additionally, they employed a transfer learning approach, utilizing eight pre-trained deep learning models to classify two breast cancer datasets. Among these models, ResNet50, optimized with the Adam optimizer, achieved the highest classification accuracy of 92.4%. Meanwhile, VGG16 demonstrated the highest area under the curve (AUC) performance, attaining a value of 0.97. Xiao et al. (2018) conducted an analysis using the breast ultrasound image dataset to evaluate the performance of InceptionV3, XceptionNet, and ResNet50. Based on their findings, they proposed a simplified breast cancer diagnosis model consisting of three convolutional layers. The dataset comprised 2,058 images, with 1,370 categorized as benign and 688 as malignant. Their experimental results indicated that InceptionV3 yielded the highest classification accuracy, achieving 85.13%. Xiaofeng et al. (Qi et al., 2022) developed a mobile phone-based methodology for diagnosing breast cancer utilizing ultrasound images. Their system comprises three subsystems: DeepRec, DeepCls, and DeepAti. DeepRec: Mitigates noise and reconstructs high-fidelity images utilizing autoencoders and GANs. DeepCls: Utilizes Convolutional Neural Networks based on pre-trained Inception-v3 to classify images as malignant or non-malignant. DeepAti: Identifies anomalies to minimize false negatives with Generative Adversarial Networks (GANs). The research employed an extensive dataset of 18,225 breast ultrasound images and 2,416 reports from three hospitals, exhibiting a good diagnostic performance with 94.51% accuracy for high-quality photos and 89.34% accuracy for low-quality images. Khan et al. (2019) proposed a novel framework for breast cancer identification and classification using transfer learning. Their approach leveraged GoogLeNet, VGG, and ResNet architectures to extract features, which were then combined and fed into a fully connected layer for classification. Moon et al. (2020) conducted a study aimed at detecting breast cancer using ultrasound imaging. They utilized two independent datasets and integrated multiple CNN models. Their methodology achieved an accuracy of 91.10% on the first dataset and 94.62% on the second, demonstrating the effectiveness of their approach. The authors in (Ye et al., 2021) prepared a gold standard dataset for their study, which included 910 benign and 934 malignant B-mode breast ultrasound images, comprising 110 triple-negative (TNBC) and 824 non-triple-negative (NTNBC) cases. A pretrained ResNet50 DCNN was employed for the analysis. The results demonstrated that the mean area under the receiver operating characteristic curve (AUC) for distinguishing malignant from benign cases was 0.9789 (benign vs TNBC) and 0.9689 (benign vs NTNBC), whereas for distinguishing TNBC from NTNBC breast cancer was 0.90, with an accuracy of 88.9%, sensitivity of 87.5%, and specificity of 90.0%.

## 3 Materials and methods

**FIGURE 1:**
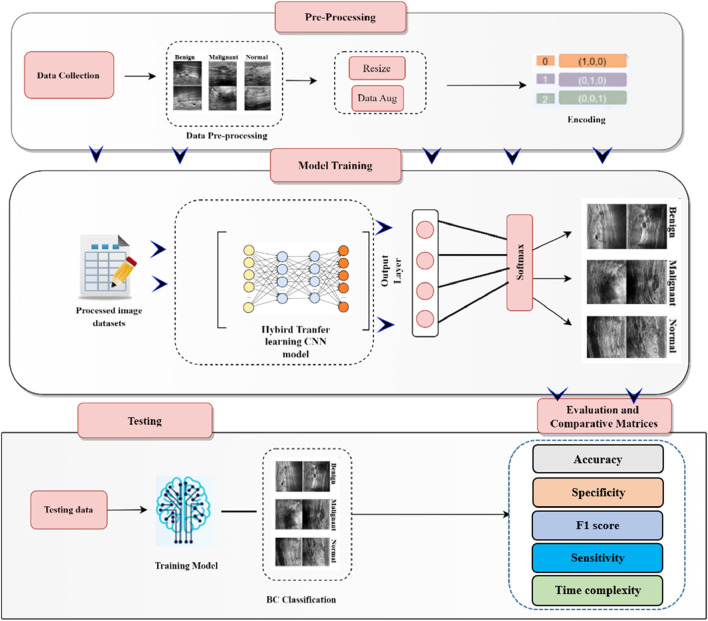
The block diagram of breast cancer detection framework using the proposed model.

This section outlines the comprehensive process of breast cancer detection, encompassing data collection, pre-processing (including data augmentation), model training, and classification. The overall workflow of the developed breast cancer detection and classification model is illustrated in Figure 1.

### 3.1 Data collection

In this study, we utilized two publicly available breast cancer databases. The motivation for incorporating both datasets is twofold: (1) to expand the size of the training dataset, thereby mitigating the risk of bias and overfitting, and (2) to include three distinct classes—normal, benign, and malignant. The integration of these datasets is expected to enhance the model’s accuracy and improve its overall effectiveness.

**FIGURE 2:**
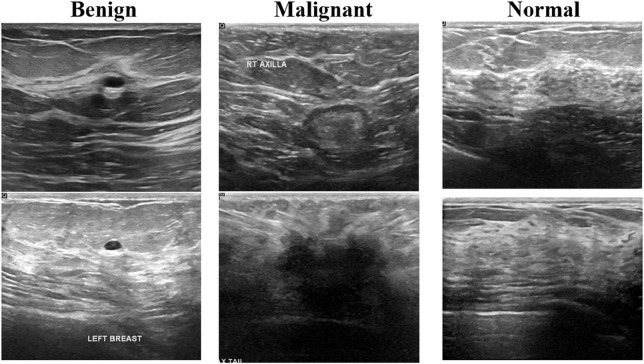
Ultrasound images of breast cancer from the selected dataset.

The first one is the breast cancer ultrasound scans BUSI (Al-Dhabyani et al., 2020). This dataset has been previously used in a published study and was compiled in 2018 from 600 distinct female subjects aged between 25 and 75 years. It comprises a total of 780 ultrasound images, categorized as follows: 133 normal, 437 benign, and 210 malignant cases. Figure 2 presents an example of an ultrasound image from this dataset. Each image has a resolution of 500 × 500 pixels and is stored in the PNG file format.

The second dataset is the Breast Ultrasound Image dataset, as described in (Paulo, 2017). This dataset comprises 250 breast cancer ultrasound images, including 100 benign and 150 malignant cases. A summary of these breast cancer datasets is presented in Table 1. The integration of these datasets enhances model performance but also introduces challenges.

**TABLE 1:** Detailed description of the two BC datasets.

Dataset	Normal	Benign	Malignant	Total
BUSI (Al-Dhabyani et al., 2020)	133	437	210	780
Breast Ultrasound Image (Paulo, 2017)	-	100	150	250
Total	133	537	360	1,030

Related to imaging conditions, device specifications, and patient demographics. Variations in ultrasound machines and imaging protocols could impact consistency, however, preprocessing techniques such as contrast normalization and intensity standardization effectively minimize these discrepancies. Additionally, while the BUSI dataset includes patient age (25–75 years), the second dataset lacks demographic details, making direct comparisons difficult. Despite this, the diverse data sources improve generalizability. Furthermore, differences in class distribution—BUSI including normal, benign, and malignant cases while the second dataset lacks normal images—were addressed through balanced sampling and data augmentation. These steps ensure a more robust and unbiased model, ultimately improving its reliability in real-world applications.

### 3.2 Pre-processing and data augmentation

The normalization step is essential before feeding images into the CNN model. This process ensures that both datasets are resized to match the input dimensions of the deep learning models. Deep learning algorithms require large amounts of data for effective training and optimal performance. However, in domains with limited data availability, training deep models becomes challenging. To address this issue, a data augmentation strategy was employed. In the training set, images were randomly shifted up to 30 pixels horizontally and vertically and rotated within a range of −30 to 30°. Additionally, image scaling was applied by randomly adjusting the size within the range of [0.9, 1.1]. This augmentation technique helped generate diverse training samples, improving the model’s generalization and preventing overfitting to the majority class, thereby enhancing the overall classification performance.

### 3.3 Deep learning model

Traditional machine learning approaches involve a sequence of steps, including pre-processing, feature extraction, and feature selection, to achieve classification. The effectiveness of these methods heavily relies on the quality of the selected features, which may not always be optimal for class discrimination. In contrast, deep learning (DL) enables automatic feature extraction tailored to specific tasks (Mendonça et al., 2023). CNNs represent a specialized subset of deep neural networks designed for analyzing visual data. CNNs process an input image by assigning weights to different components, allowing them to distinguish between various elements within the image. Due to their ability to learn hierarchical features directly from raw data, CNNs have demonstrated exceptional accuracy in image classification and recognition tasks (Ba Mahel et al., 2024; Alabdulhafith et al., 2024; Ba Mahel and Kalinichenko, 2024; Ba Mahel and Kalinichenko, 2022; Ba Mahel et al., 2022; Nemirko et al., 2024; Mahel et al., 2024).

### 3.4 Transfer learning models

**FIGURE 3:**
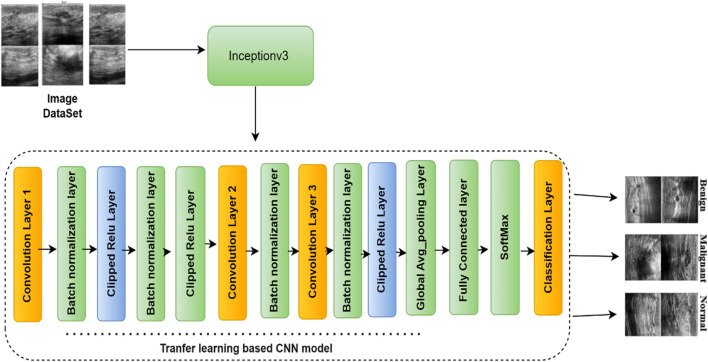
The block diagram of the proposed model.

Deep CNN techniques continue to be widely used due to their ability to provide innovative solutions for detection and classification tasks. However, a common challenge with deep CNN models is their reliance on large amounts of training data, which may not always be readily available. Acquiring and annotating large datasets is often time-consuming and resource-intensive. To address this limitation, transfer learning (TL) has emerged as an effective approach (Kim et al., 2022). Transfer learning involves pre-training CNN models on large datasets and then fine-tuning them for a smaller, domain-specific dataset. This approach significantly reduces training time and computational cost while improving model performance, even with limited training data. Since the pre-trained model has already learned fundamental features, it requires less data to achieve high accuracy. One widely used dataset for pre-training deep learning models is the ImageNet dataset (Russakovsky et al., 2015), which contains over 15 million images categorized into more than 22,000 classes. Many state-of-the-art CNN architectures have been trained on ImageNet and are frequently utilized for transfer learning applications. In this study, rather than training the model from scratch, the BUSI dataset was used for fine-tuning and training the final classification model. Transfer learning is one of the most effective techniques for addressing computer vision challenges, especially when data availability is limited. Figure 3 illustrates the fine-tuning process of nine different CNN models for breast cancer detection and classification. Additionally, Table 2 provides a detailed overview of the number of parameters in millions and layers associated with each CNN architecture used in this research.

**TABLE 2:** The parameters and layers of the pre-train model for breast cancer detection.

Methods	Number of layers	Size	Parameters (M)
AlexNet	8	227 Mb	60 M
ResNet-101	101	171 MB	44 M
Inception-v3	42	93 MB	27 M
VGG-16	50	96 MB	25.6 M
GoogLeNet	22	27 MB	7 M

#### 3.4.1 XceptionNet

Xception is an advanced DL architecture that builds upon and improves the Inception model. Instead of using traditional convolutional layers, Xception employs depthwise separable convolutions, which decouple spatial and cross-channel correlations. This separation allows for more efficient feature extraction and computational savings compared to standard CNNs. Unlike conventional CNNs that blend spatial and cross-channel relationships, Xception processes them independently. This architectural enhancement improves model performance and robustness, making Xception more effective than its predecessor, Inception. The Xception model consists of 36 convolutional layers, which are grouped into 14 distinct modules. After removing the initial and final layers, residual connections link each consecutive layer, enhancing gradient flow and training stability. The model first captures cross-channel relationships within the input image and then translates them into spatial correlations within each output channel. This process is followed by depthwise 1 × 1 convolutions, which refine feature representation while maintaining computational efficiency. These architectural innovations contribute to Xception’s superior performance across various image classification and recognition tasks.

#### 3.4.2 GoogLeNet

GoogLeNet has 144 layers and uses Inception modules with four parallel branches (1 × 1, 3 × 3, 5 × 5 convolutions, and max pooling). It applies ReLU activation in all convolutions. 1 × 1 convolutions reduce parameters, improving efficiency. The architecture minimizes parameters from 60 million to ∼4 million, enhancing computational performance without losing accuracy.

#### 3.4.3 ShuffleNet

The ShuffleNet model, comprising 50 layers, processes input images of 224 × 224 pixels. It extracts 544 deep features through global average pooling, enabling advanced image representation. Pre-trained on the ImageNet dataset, ShuffleNet efficiently classifies new tasks by leveraging learned features from large-scale image data.

#### 3.4.4 AlexNet

The AlexNet architecture consists of eleven layers, designed to enhance feature extraction. While its depth improves learning, numerous parameters can impact performance. The first layer is a convolutional layer, followed by max pooling and normalization layers. The network concludes with a SoftMax layer for classification.

#### 3.4.5 SqueezeNet

The SqueezeNet model consists of 18 layers and processes input images of 227 × 227 pixels. Despite having fewer parameters, it achieved high accuracy on ImageNet. High-level features were extracted using activations from the topmost fully connected layer, interpreting input images as 1,000 deep features.

#### 3.4.6 Proposed model (InceptionNetV3)

The convolution process constitutes the most critical phase within a neural network that incorporates convolutional layers. Typically, convolution algorithms that employ larger spatial filters demand substantial computational resources. The adoption of the Inception module represents a pivotal advancement in mitigating these costs. By leveraging optimally efficient local sparse structures, the computational expense associated with the Inception module is significantly reduced. The design of the Inception block predominantly depends on statistical analysis of layer correlations during the layer-by-layer construction process. Filter banks are formed from interconnected layers, and the ultimate outcomes may be achieved by concatenating multiple large filter banks within a single region. However, these filter banks introduce patch alignment challenges, which can be addressed by employing smaller filter sizes, such as 1 × 1, 3 × 3, and 5 × 5. Furthermore, in the sequence of computations for dimensionality reduction, the 1 × 1 convolution precedes the 3 × 3 and 5 × 5 convolutions. The internal architecture of an Inception module, illustrated in Figure 3, serves as the foundation for InceptionNet. To extract features from images across varying spatial resolutions, input images are convolved with kernels of different sizes, such as 3 × 3 and 5 × 5. In the final stage, the activation maps derived from these parallel computations are concatenated depth-wise to produce the desired output.

Additionally, the Inception block depicted in Figure 3 operates with reduced dimensions. The input is processed through four parallel convolutional pathways, consisting of three scaled convolutions and one pooling operation. The first pathway involves a 1 × 1 convolution followed by a 5 × 5 convolution, while the second pathway comprises a 1 × 1 convolution followed by a 3 × 3 convolution (Das et al., 2020). The third pathway employs pooling prior to a 1 × 1 convolution, and the fourth pathway follows a similar procedure (Szegedy et al., 2016). The output of the Inception block is generated by concatenating the filtered results of these four convolutional pathways, resulting in extensive spatial filtering that accounts for layer correlations.

The InceptionNetV3 model, pre-trained on the ImageNet dataset, comprises 48 deep layers that utilize the ReLU activation function and integrate 1 × 1, 3 × 3, and 5 × 5 convolutional layers (Kokkalla et al., 2021; Szegedy et al., 2015). The model’s upper structure has been modified to enable binary classification. To effectively constrain the number of trainable parameters within the convolutional layers, these parameters were designated as non-trainable, reducing the total from 22,982,626 to 1,179,842. Despite its depth, the architecture of InceptionNetV3 substantially lowers the number of training parameters compared to earlier neural networks. For instance, VGG16 encompasses approximately 90 million parameters but offers considerably less depth than InceptionNetV3. Greater depth enhances model accuracy by enabling the capture of finer details. The InceptionV3 framework underpins the enhanced InceptionNetV3 architecture. Utilizing a pre-trained model, rather than constructing a new network from scratch, is recommended. InceptionNetV3 was selected as the foundation due to its demonstrated efficacy with biomedical data and the benefits derived from its pre-trained knowledge, aligning with the goal of optimizing model performance. In the customized version of the InceptionNetV3 model, the top four layers have been removed, transforming it into an InceptionNetV3 variant. These layers have been replaced with fifteen additional layers. The architecture of this tailored InceptionNetV3 model is detailed in Figure 3. The sequential arrangement of layers is critical in hybrid architectures and any subsequent advancements in CNN designs employed in DL. Comprehensive details regarding the properties of these additional layers are provided in Table 3. These custom layers improve performance by addressing common DL challenges. Clipped ReLU Activation prevents vanishing or exploding gradients, ensuring stable training and faster convergence. Group Convolution reduces parameters and computational costs by partitioning input channels, which helps reduce overfitting and increase efficiency. Together, these layers enhance stability, reduce complexity, and improve model performance without compromising predictive power.

**TABLE 3:** Additional parameters and layers information of the proposed model.

S.No	Name of layer	Filter size	Filter/Neurons	Epsilon
1	Conv_1	1 × 1	960	
2	BatchNormalization_1			0.001
3	Clipped_ReLU_Activation_Layer_1			
4	Group_Conv	3 × 3	960	
5	BatchNormalization_2			0.001
6	Clipped_ReLU_Activation_Layer_2			0.001
7	Conv_2	1 × 1	320	
8	BatchNormalization_3			0.001
9	Conv_3	1 × 1	1,280	
10	BatchNormalization_4			0.001
11	Clipped_ReLU_Activation_Layer_3			0.001
12	GlobalAvg_ Pooling_Layer		64	
13	FullyConnected_Layer		3	
14	Softmax_Layer			
15	FullyConnected_Classification_Layer with 3 output neurons (Softmax)		3	

### 3.5 Hyperparameters and experimental settings

Setting hyperparameters before training is essential, as they significantly influence the learning process and model performance. Various methods exist to determine the optimal values. For training, breast cancer ultrasound images were split using a 70/30 ratio. The number of training samples tallied in a single forward and backward pass is called the batch size. As the batch size is increased, there is a corresponding increase in the amount of memory space that is necessitated. Due to hardware constraints, we set the batch size to 10 images. An epoch represents one complete pass through the training dataset. With transfer learning and model training, we set 50 epochs, requiring 82 iterations per epoch to complete. Furthermore, we trained both the pre-trained and modified models using a learning rate of 0.001. The detailed parameter specifications for both the customized model and pre-trained models are presented in Table 4. We employed stochastic gradient descent (SGD) as the optimization algorithm for training the proposed model and conducted our experiments in Python 3.8 and TensorFlow 2.9.

**TABLE 4:** The hyperparameters of the proposed model.

Parameters	Values given
Validation frequency	82
Learning rate	0.001
Optimization algorithm	SGDM
Iterations per epoch	82
Shuffle	Every epoch
Verbose	false
Batch size	10
Maximum epochs	50
Activation function	Leaky ReLu + ReLu

## 4 Results and discussion

To assess the proposed model, we employ the metrics outlined in Equations 1–4. The “True Positive” (TP) concept pertains to positive data that has been accurately predicted and assessed. The diagonal contains the most prominent values.

A true negative (TN) is when a test or diagnostic procedure correctly identifies the absence of a particular condition or attribute. A false positive (FP) refers to data that should have been classified as negative but are erroneously identified as positive during evaluation. The summation is computed by adding up all the values in the column corresponding to each class, excluding TP. One instance of a false negative can be observed in interpreting positive information as having a detrimental effect. The summation encompasses all the values within the row corresponding to each class, excluding TP.

$$Accuracy=\frac{TP+TN}{TP+FP+TN+FN}$$ (1)

$$precision=\frac{TP}{TP+FP}$$ (2)

$$Recall=\frac{TP}{TP+FN}$$ (3)

$$F1-Score=2\cdot \frac{\left(precision\cdot recall\right)}{\left(precision+recall\right)}$$ (4)

**FIGURE 4:**
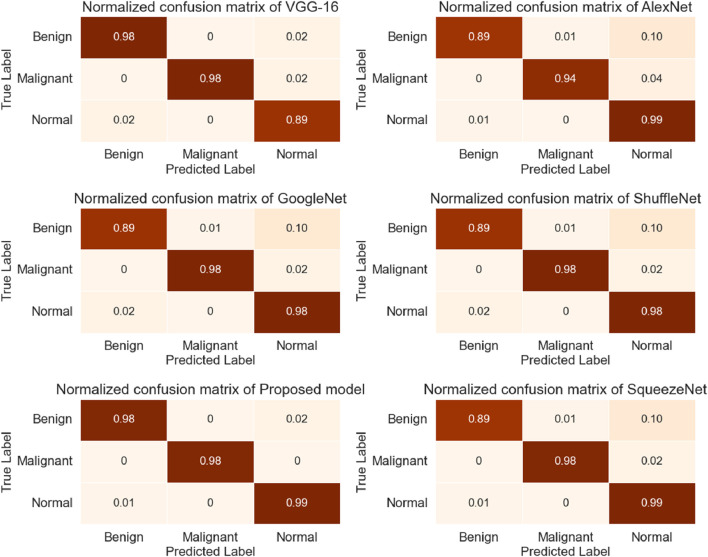
The confusion matrix of the CNN-based transfer learning models.

During this study, the dataset was split into training and testing, with 70% of the data used for model training and 30% used for testing. The proposed research used DL-based classification strategies such as Alex Net, VGG-16, SqueezeNet, Shufflenet, and modified InceptionNet-v3. The confusion matrices generated by the various pre-trained models, as well as the proposed customized model, are presented in Figure 4. It was observed that InceptionNet-V3 achieved the highest accuracy at 99%, while ShuffleNet obtained the lowest accuracy at 95%, followed by GoogLeNet with the second-lowest accuracy. Furthermore, the results exceeded the performance of the pre-trained models. Table 5 presents the classification results for each model. In terms of accuracy, precision, recall, and F1-score, the customized InceptionNet-V3 model outperformed all pre-trained models.

**TABLE 5:** Pre-trained model experimental results.

Model	Accuracy (%)	Precision (%)	Recall (%)	F1-score (%)
Modified InceptionNet-v3	99.10	98.90	99.05	98.80
GoogLeNet	95.70	95.50	95.40	95.20
AlexNet	96.10	95.70	95.80	95
Shufflenet	95	94.60	94.70	94.30
SqueezeNet	97.10	97.05	96.90	96
VGG-16	96.40	96.10	95.90	95.80

**FIGURE 5:**
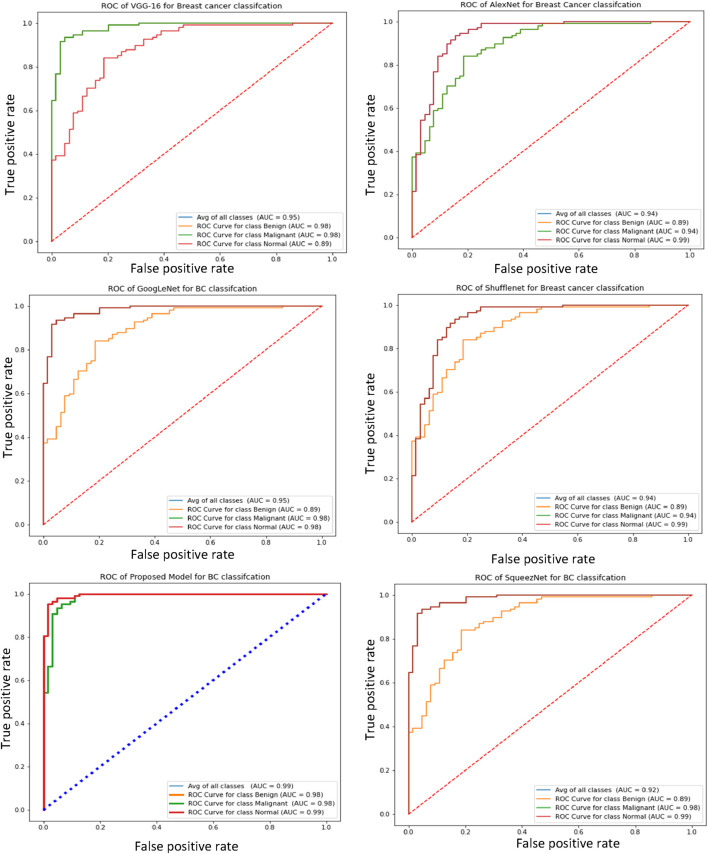
The ROC curves of the proposed and pre-trained transfer learning models.

Furthermore, the receiver operating characteristic (ROC) curve is a crucial metric for breast cancer detection, as it provides a comparative evaluation of the true negative rate (TNR) and true positive rate (TPR). Figure 5 illustrates the ROC curves of the proposed and pre-trained models, effectively demonstrating the relationship between true positives and true negatives. Notably, the proposed model exhibits higher average ROC values compared to the pre-trained transfer learning models, indicating superior performance.

To conduct a comprehensive evaluation and analysis of the proposed model, a confusion matrix is employed to compute the true negative rate (TNR), true positive rate (TPR), and Matthews correlation coefficient (MCC). The success rates for TPR, TNR, and MCC are presented in Table 6, with the proposed model achieving 99.1%, 99.2%, and 0.99, respectively. These results demonstrate the model’s superior performance. Furthermore, the proposed model has been compared against several benchmark algorithms. Table 7 presents the comparative results with the most recent benchmarks. The proposed approach outperforms existing methods across key evaluation metrics, including accuracy (Acc), precision (Pres), sensitivity (Sens), specificity (Spec), and others.

**TABLE 6:** The TPR, TNR, and MCC values.

Model	TPR (%)	TNR (%)	MCC (%)
Modified InceptionNet-v3	99	98	99
GoogLeNet	95	95	95
AlexNet	96	95	95
Shufflenet	95	94	94
SqueezeNet	97	97	96
VGG-16	95	95	95

**TABLE 7:** A comparative study of the proposed with recent ML/DL models of breast cancer detection.

References	Model	Dataset	Purpose	Accuracy (%)
Latha et al. (2024)	SLIC-based ROI extraction + SVM	BUSI	Classify breast tumors in ultrasound images	88%
Zhang et al. (2022)	XGBoost-based ML model	952 patients’ ultrasound images	Predict sentinel lymph node metastasis in breast cancer patients	84.6%
Lyu et al. (2023)	Radiomics model with random forest	302 small breast masses’ ultrasound images	Differentiate minimal breast cancer from small benign breast masses	AUC: 79.3%
Rakha et al. (2024)	MobileNetV2 combined with CBAM	Combined dataset from two ultrasound image sources	Classify breast cancer ultrasound images	93.00%
Zhuang et al. (2021)	Image decomposition and fusion with adaptive multi-model spatial feature fusion	Collected 1,328 BUSI	Classify breast tumors in ultrasound images	95.48%
Pang et al. (2021)	Semi-supervised GAN-based radiomics model	Collected 1,447 BUSI	Data augmentation for breast ultrasound mass classification	90.41%
This work	Modified InceptionNet-v3	BUSI	Breast cancer image classification (benign, malignant, normal)	99.10%

### 4.1 Explainability analysis using Grad-CAM

**FIGURE 6:**
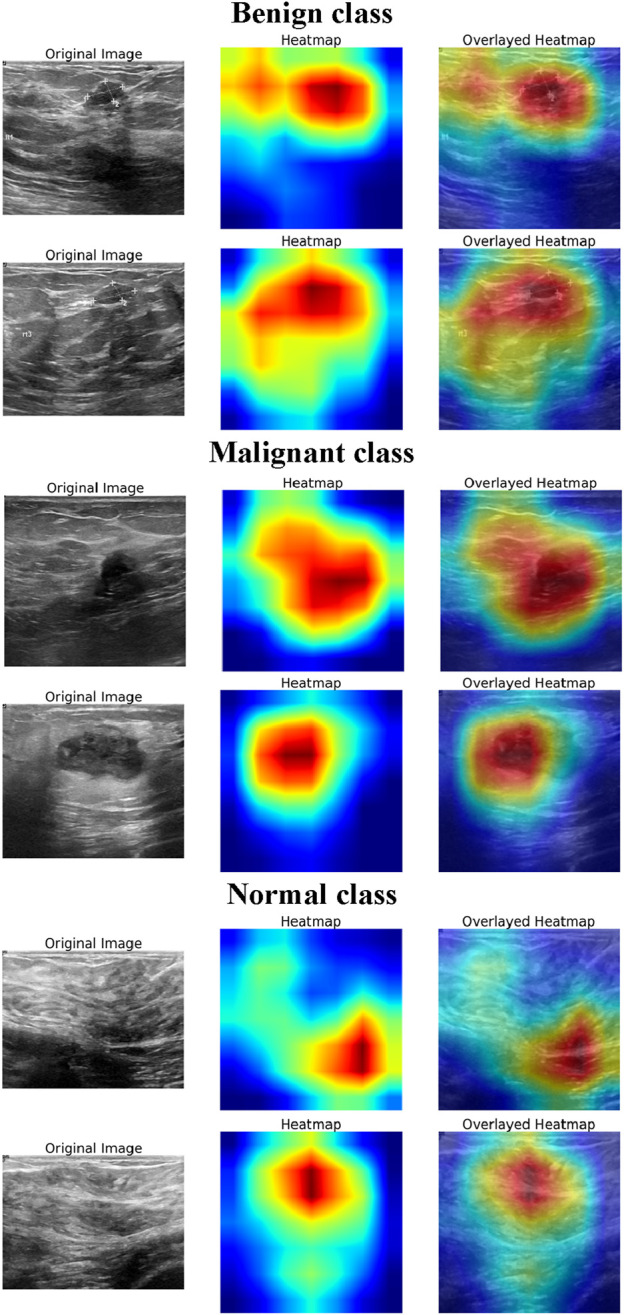
Grad-CAM visualization for model explainability in BUSI classification.

To enhance the interpretability of the DL model’s decision-making process, Grad-CAM visualizations (Selvaraju et al., 2016) were generated for normal, benign, and malignant BUSI. Figure 6 presents the original ultrasound images, the corresponding Grad-CAM heatmaps, and the overlayed heatmaps for each class, illustrating the model’s focus during classification. For the normal class, the heatmaps exhibit minimal and dispersed activation, indicating that the model does not focus on specific regions but instead confirms the absence of suspicious structures. The activations are primarily distributed across homogeneous tissue areas, suggesting that the model correctly identifies the lack of distinct lesion-like features. This highlights the model’s ability to distinguish between normal breast tissue and pathological cases effectively. In benign cases, the heatmaps show moderate activation over well-circumscribed, hypoechoic regions, which are characteristic of benign breast lesions. The model’s attention is distributed over the lesion area but lacks sharply concentrated activation, suggesting that it relies on structural attributes such as shape, margins, and internal echotexture to make its predictions. For malignant lesions, the model exhibits strong and localized activations in the central and peripheral regions of the lesion. The heatmaps reveal an intense focus on irregular, heterogeneous areas, which are the indicators of malignancy in BUSI. The sharp, well-defined areas of attention indicate that the model captures crucial morphological characteristics such as irregular contours, and echogenic halo regions. Thus, the explainability analysis demonstrates that the model effectively leverages relevant diagnostic features for classification, aligning with clinical knowledge. The distinct activation patterns observed for normal, benign, and malignant classes suggest that the model accurately differentiates between them, reinforcing its potential for assisting in breast cancer diagnosis.

### 4.2 Limitations and future directions

This research’s drawback is that only a restricted quantity of images in the breast cancer ultrasound imaging dataset is available in public. This affects how well DL models perform. This research has the potential to be improved further by including additional images and datasets. In addition, the outlook for the future study might concentrate on answering therapeutically related questions. The effective development of improved deep learning algorithms may assist radiologists and oncologists in accurately detecting breast cancer using MRI and CT scans.

## 5 Conclusion

This study evaluated five pre-trained DL models for breast cancer detection using ultrasound images. The experimental results demonstrated that the modified InceptionNet-V3 model achieved the highest accuracy of 99.10%, outperforming other models, while GoogLeNet obtained the lowest accuracy of 95%. By optimizing the best-performing model, we further enhanced classification performance, highlighting the effectiveness of transfer learning in medical image analysis. The findings provide valuable insights for researchers and practitioners in selecting optimal models for breast cancer diagnosis.

Future work should focus on extending the dataset to improve model generalization across diverse imaging conditions and patient demographics. Additionally, exploring lightweight architectures will facilitate real-time deployment in healthcare settings.

## Data Availability

Publicly available datasets were analyzed in this study. This data can be found here: https://data.mendeley.com/datasets/wmy84gzngw/1 and https://scholar.cu.edu.eg/?q=afahmy/pages/dataset.

## References

[B1] Alabdulhafith M. Ba Mahel A. S. Samee N. A. Mahmoud N. F. Talaat R. Muthanna M. S. (2024). Automated wound care by employing a reliable U-Net architecture combined with ResNet feature encoders for monitoring chronic wounds. Front. Med. 11, 1310137. 10.3389/fmed.2024.1310137 PMC10865496 38357646

[B2] Al-Dhabyani W. Gomaa M. Khaled H. Fahmy A. (2020). Dataset of breast ultrasound images. Data Brief 28, 104863. 10.1016/j.dib.2019.104863 31867417 PMC6906728

[B3] Allaoua Chelloug S. Alkanhel R. Muthanna M. S. A. Aziz A. Muthanna A. (2023). MULTINET: a multi-agent drl and EfficientNet assisted framework for 3D plant leaf disease identification and severity quantification. IEEE Access 11, 86770–86789. 10.1109/access.2023.3303868

[B4] Ara S. Das A. Dey A. (2021). “Malignant and benign breast cancer classification using machine learning algorithms,” in 2021 international conference on artificial intelligence (Islamabad, Pakistan: ICAI), 97–101. 10.1109/ICAI52203.2021.9445249

[B5] Ba Mahel A. S. Cao S. Zhang K. Chelloug S. A. Alnashwan R. Muthanna M. S. (2024). Advanced integration of 2DCNN-GRU model for accurate identification of shockable life-threatening cardiac arrhythmias: a deep learning approach. Front. Physiology 15, 1429161. 10.3389/fphys.2024.1429161 PMC11272599 39072217

[B6] Ba Mahel A. S. Harold N. Solieman H. (2022). “Arrhythmia classification using alexnet model based on orthogonal leads and different time segments,” in 2022 conference of Russian young researchers in electrical and electronic engineering (ElConRus) (Saint Petersburg: Russian Federation), 1312–1315. 10.1109/ElConRus54750.2022.9755708

[B7] Ba Mahel A. S. Kalinichenko A. N. (2022). “Classification of cardiac cycles using a convolutional neural network,” in 2022 conference of Russian young researchers in electrical and electronic engineering (ElConRus) (Saint Petersburg: Russian Federation), 1466–1469. 10.1109/ElConRus54750.2022.9755490

[B8] Ba Mahel A. S. Kalinichenko A. N. (2024). “Classification of arrhythmia using parallel channels and different features,” in 2024 conference of young researchers in electrical and electronic engineering (ElCon) (Saint Petersburg: Russian Federation), 1007–1010. 10.1109/ElCon61730.2024.10468316

[B9] Borah N. Varma P. S. P. Datta A. Kumar A. Baruah U. Ghosal P. (2022). “Performance analysis of breast cancer classification from mammogram images using vision transformer,” in 2022 IEEE Calcutta conference (CALCON) (IEEE), 238–243.

[B10] Byra M. Jarosik P. Szubert A. Galperin M. Ojeda-Fournier H. Olson L. (2020). Breast mass segmentation in ultrasound with selective kernel U-Net convolutional neural network. Biomed. signal Process. control 61, 102027. 10.1016/j.bspc.2020.102027 34703489 PMC8545275

[B11] Chahal P. K. Pandey S. Goel S. (2020). A survey on brain tumor detection techniques for MR images. Multimedia Tools Appl. 79 (29), 21771–21814. 10.1007/s11042-020-08898-3

[B18] Chiu H.-J. Li T.-H. S. Kuo P.-H. (2020). “Breast cancer–detection system using PCA, multilayer perceptron, transfer learning, and support vector machine,” in IEEE Access 8, 204309–204324. 10.1109/ACCESS.2020.3036912

[B12] Das D. Santosh K. C. Pal U. (2020). Truncated inception net: COVID-19 outbreak screening using chest X-rays. Phys. Eng. Sci. Med. 43 (3), 915–925. 10.1007/s13246-020-00888-x 32588200 PMC7315909

[B13] Din N. M. U. Dar R. A. Rasool M. Assad A. (2022). Breast cancer detection using deep learning: datasets, methods, and challenges ahead. Comput. Biol. Med. 149, 106073. 10.1016/j.compbiomed.2022.106073 36103745

[B14] Dubey M. Kumar S. (2024). “A model for the diagnosis and prognosis of breast cancer based on fuzzy expert system,” in Mathematical sciences and applications (CRC Press), 30–36.

[B15] Elton D. C. Turkbey E. B. Pickhardt P. J. Summers R. M. (2022). A deep learning system for automated kidney stone detection and volumetric segmentation on non‐contrast CT scans. Med. Phys. 49, 2545–2554. 10.1002/mp.15518 35156216 PMC10407943

[B16] Eroğlu Y. Yildirim M. Çinar A. (2021). Convolutional Neural Networks based classification of breast ultrasonography images by hybrid method with respect to benign, malignant, and normal using mRMR. Comput. Biol. Med. 133, 104407. 10.1016/j.compbiomed.2021.104407 33901712

[B17] Ghosh D. Kumar A. Ghosal P. Chowdhury T. Sadhu A. Nandi D. (2020). “Breast lesion segmentation in ultrasound images using deep convolutional neural networks,” in 2020 IEEE Calcutta conference (CALCON) (IEEE), 318–322.

[B19] Hagos B. Yeman A. Mérida G. Teuwen J. (2018). “Improving breast cancer detection using symmetry information with deep learning,” in Image analysis for moving organ, breast, and thoracic images (Cham: Springer), 90–97.

[B20] He K. Zhang X. Ren S. Sun J. (2016). “Deep residual learning for image recognition,” in Proceedings of the IEEE conference on computer vision and pattern recognition, USA, 18-20 June 1996, 770–778.

[B21] Hirra I. Ahmad M. Hussain A. Ashraf M. U. Saeed I. A. Qadri S. F. (2021). Breast cancer classification from histopathological images using patch-based deep learning modeling. IEEE Access 9, 24273–24287. 10.1109/access.2021.3056516

[B22] Huang G. Liu Z. Van Der Maaten L. Weinberger K. Q. (2017). “Densely connected convolutional networks,” in Proceedings of the IEEE conference on computer vision and pattern recognition, USA, 17-19 June 1997, 4700–4708.

[B23] Iandola F. N. Han S. Moskewicz M. W. Ashraf K. Dally W. J. Keutzer K. (2016). SqueezeNet: AlexNet-level accuracy with 50x fewer parameters and <0.5MB model size. ArXiv. 10.48550/arXiv.1602.07360

[B24] Inan H. Khan M. S. Alam F. I. Hasan R. (2022). Deep integrated pipeline of segmentation guided classification of breast cancer from ultrasound images. Biomed. Signal Process. Control 75, 103553. 10.1016/j.bspc.2022.103553

[B25] Jebarani P. E. Umadevi N. Dang H. Pomplun M. (2021). A novel hybrid K-means and GMM machine learning model for breast cancer detection. IEEE Access 9, 146153–146162. 10.1109/access.2021.3123425

[B26] Khan S. U. Islam N. Jan Z. Din I.Ud Rodrigues J. J. P. C. (2019). A novel deep learning based framework for the detection and classification of breast cancer using transfer learning. Pattern Recognit. Lett. 125, 1–6. 10.1016/j.patrec.2019.03.022

[B27] Khuriwal N. Mishra N. (2018). “Breast cancer diagnosis using deep learning algorithm,” in 2018 international conference on advances in computing, communication control and networking (ICACCCN) (IEEE).

[B28] Kim H. E. Cosa-Linan A. Santhanam N. Jannesari M. Maros M. E. Ganslandt T. (2022). Transfer learning for medical image classification: a literature review. BMC Med. Imaging 22, 69. 10.1186/s12880-022-00793-7 35418051 PMC9007400

[B29] Kokkalla S. Kakarla J. Venkateswarlu I. B. Singh M. (2021). Three-class brain tumor classification using deep dense inception residual network. Soft Comput. 25 (13), 8721–8729. 10.1007/s00500-021-05748-8 33897297 PMC8051839

[B30] Krizhevsky A. Sutskever I. Hinton G. E. (2012). Imagenet classification with deep convolutional neural networks. Commun. ACM 60 6, 84–90. 10.1145/3065386

[B31] Latha M. Kumar P. S. Chandrika R. R. Mahesh T. R. Kumar V. V. Guluwadi S. (2024). Revolutionizing breast ultrasound diagnostics with EfficientNet-B7 and Explainable AI. BMC Med. Imaging 24, 230. 10.1186/s12880-024-01404-3 39223507 PMC11367906

[B32] Loizidou K. Elia R. Pitris C. (2023). Computer-aided breast cancer detection and classification in mammography: a comprehensive review. Comput. Biol. Med. 153, 106554. 10.1016/j.compbiomed.2023.106554 36646021

[B33] Lyu S. Zhang M. Zhang B. Zhu J. Gao L. Qiu Y. (2023). The value of radiomics model based on ultrasound image features in the differentiation between minimal breast cancer and small benign breast masses. J. Clin. Ultrasound 51 (9), 1536–1543. 10.1002/jcu.23556 37712556

[B34] Madej-Czerwonka B. Korga-Plewko A. Czerwonka M. (2022). Modern breast cancer diagnostic methods. Curr. Issues Pharm. Med. Sci. 35 (1), 1–5. 10.2478/cipms-2022-0001

[B35] Mahel A. S.Ba Alotaibi F. M. G. Rao N. (2024). “The role of synthetic data in mitigating imbalance in deep-learning-based arrhythmia classification: a comparative study,” in Sixth international conference on image, video processing, and artificial intelligence (IVPAI 2024) (SPIE), 13225, 16. 10.1117/12.3046225

[B36] Masud M. Eldin Rashed A. E. Hossain M. S. (2022). Convolutional neural network-based models for diagnosis of breast cancer. Neural Comput. Appl., 34, 11383–11394. 10.1007/s00521-020-05394-5 33052172 PMC7545025

[B37] Mendonça M. O. Netto S. L. Diniz P. S. Theodoridis S. (2023). Machine learning: review and trends. Signal Process. Mach. Learn. Theory, 869–959. 10.1016/B978-0-32-391772-8.00019-3

[B38] Mondal M. Ghosal P. Kumar A. Nandi D. (2023). “A modified microwave based system design for early-stage breast cancer detection,” in International conference on advanced computational and communication paradigms (Singapore: Springer Nature Singapore), 251–258.

[B39] Moon W. K. Lee Y.-W. Ke H.-H. Su H. L. Huang C.-S. Chang R.-F. (2020). Computer‐aided diagnosis of breast ultrasound images using ensemble learning from convolutional neural networks. Comput. methods programs Biomed. 190, 105361. 10.1016/j.cmpb.2020.105361 32007839

[B40] Nemirko A. P. Ba Mahel A. S. Manilo L. A. (2024). Recognition of life-threatening arrhythmias by ECG scalograms. Comput. Opt. 48 (1), 149–156. 10.18287/2412-6179-co-1354

[B41] Pang T. Wong J. H. D. Ng W. L. Chan C. S. (2021). Semi-supervised GAN-based radiomics model for data augmentation in breast ultrasound mass classification. Comput. Methods Programs Biomed. 203, 106018. 10.1016/j.cmpb.2021.106018 33714900

[B42] Pathak V. Singh K. Ahmed A. Dhoot A. (2020). Efficient and compressive IoT based Health care system for Parkinson’s disease patient. Procedia Comput. Sci. 167, 1046–1055. ISSN 1877-0509. 10.1016/j.procs.2020.03.441

[B43] Paulo S. R. (2017). Breast ultrasound image. Mendeley data. 10.17632/wmy84gzngw.1

[B44] Prusty S. Das P. Dash S. K. Patnaik S. (2023). RETRACTED: prediction of Breast cancer using integrated machine learning-fuzzy and dimension reduction techniques. J. Intelligent and Fuzzy Syst. 45 (1), 1633–1652. 10.3233/jifs-223265

[B45] Qi X. Yi F. Zhang L. Chen Y. Pi Y. Chen Y. (2022). Computer-aided diagnosis of breast cancer in ultrasonography images by deep learning. Neurocomputing 472, 152–165. 10.1016/j.neucom.2021.11.047

[B46] Rafiq A. Alkanhel R. Muthanna M. S. A. Mokrov E. Aziz A. Muthanna A. (2023). Intelligent resource allocation using an artificial ecosystem optimizer with deep learning on UAV networks. Drones 7, 619. 10.3390/drones7100619

[B47] Ragab D. Sharkas M. Al-Sharkawy M. Abukir A. (2013). “A comparison between support vector machine and artificial neural network for breast cancer detection,” in Proceedings of the 12th International Conference on Signal Process. Robot. Autom, Cambridge, UK, 17-19 June 1997 (ISPRA’13), 20–22.

[B48] Ragab D. A. Sharkas M. Marshall S. Ren J. (2019). Breast cancer detection using deep convolutional neural networks and support vector machines. PeerJ 7, e6201. 10.7717/peerj.6201 30713814 PMC6354665

[B49] Rakha M. Sulistiyo M. D. Nasien D. Ridha M. (2024). A combined MobileNetV2 and CBAM model to improve classifying the breast cancer ultrasound images. J. Appl. Eng. Technol. Sci. (JAETS) 6, 561–578. n. pag. 10.37385/jaets.v6i1.4836

[B50] Roslidar R. Rahman A. Muharar R. Syahputra M. R. Arnia F. Syukri M. (2020). A review on recent progress in thermal imaging and deep learning approaches for breast cancer detection. IEEE Access 8, 116176–116194. 10.1109/access.2020.3004056

[B51] Russakovsky O. Deng J. Su H. Krause J. Satheesh S. Ma S. (2015). ImageNet large scale visual recognition challenge. Int. J. Comput. Vis. 115 (3), 211–252. 10.1007/s11263-015-0816-y

[B52] Saber A. Mohamed S. Abo-Seida O. M. Keshk A. Chen H. (2021). A novel deep-learning model for automatic detection and classification of breast cancer using the transfer-learning technique. IEEE Access 9, 71194–71209. 10.1109/access.2021.3079204

[B53] Selvaraju R. R. Cogswell M. Das A. Vedantam R. Parikh D. Batra D. (2016). Grad-CAM: visual explanations from deep networks via gradient-based localization. ArXiv 128, 336–359. 10.1007/s11263-019-01228-7

[B54] Selvathi D. Aarthy Poornila A. (2018). “Deep learning techniques for breast cancer detection using medical image analysis,” in Biologically rationalized computing techniques for image processing applications (Cham: Springer), 159–186.

[B55] Sharma S. Kumar S. (2021). The Xception model: a potential feature extractor in breast cancer histology images classification. ICT Express 8, 101–108. 10.1016/j.icte.2021.11.010

[B56] Solanki Y. S. Chakrabarti P. Jasinski M. Leonowicz Z. Bolshev V. Vinogradov A. (2021). A hybrid supervised machine learning classifier system for breast cancer prognosis using feature selection and data imbalance handling approaches. Electronics 10 (6), 699. 10.3390/electronics10060699

[B57] Spanhol F. A. Oliveira L. S. Petitjean C. Heutte L. (2015). A dataset for breast cancer histopathological image classification. Ieee Trans. Biomed. Eng. 63 (7), 1455–1462. 10.1109/TBME.2015.2496264 26540668

[B58] Szegedy C. Liu W. Jia Y. Sermanet P. Reed S. Anguelov D. (2015). “Going deeper with convolutions,” in Proceedings of the IEEE conference on computer vision and pattern recognition, 1–9.

[B59] Szegedy C. Vincent V. Ioffe S. Shlens J. Wojna Z. (2016). “Rethinking the inception architecture for computer vision,” in Proceedings of the IEEE conference on computer vision and pattern recognition, 2818–2826.

[B60] Ting F. F. Tan Y. J. Sim K. S. (2019). Convolutional neural network improvement for breast cancer classification. Expert Syst. Appl. 120, 103–115. 10.1016/j.eswa.2018.11.008

[B61] Toğaçar M. Özkurt K. B. Ergen B. Cömert Z. (2020). BreastNet: a novel convolutional neural network model through histopathological images for the diagnosis of breast cancer. Phys. A Stat. Mech. its Appl. 545, 123592. 10.1016/j.physa.2019.123592

[B62] Xiao T. Liu L. Li K. Qin W. Yu S. Li Z. (2018). Comparison of transferred deep neural networks in ultrasonic breast masses discrimination. Biomed. Res. Int. 2018, 4605191. 10.1155/2018/4605191 30035122 PMC6033250

[B63] Ye H. Hang J. Zhang M. Chen X. Ye X. Chen J. (2021). Automatic identification of triple negative breast cancer in ultrasonography using a deep convolutional neural network. Sci. Rep. 11 (1), 20474. 10.1038/s41598-021-00018-x 34650065 PMC8517009

[B64] Zhang G. Shi Y. Yin P. Liu F. Fang Y. Li X. (2022). A machine learning model based on ultrasound image features to assess the risk of sentinel lymph node metastasis in breast cancer patients: applications of scikit-learn and SHAP. Front. Oncol. 12, 944569. PMID: 35957890; PMCID: PMC9359803. 10.3389/fonc.2022.944569 35957890 PMC9359803

[B65] Zhang X. Zhou X. Lin M. Sun J. (2017).ShuffleNet: an extremely efficient convolutional neural network for mobile devices. arXiv preprint arXiv:1707.01083v2.

[B66] Zhuang Z. Yang Z. Raj A. N. J. Wei C. Jin P. Zhuang S. (2021). Breast ultrasound tumor image classification using image decomposition and fusion based on adaptive multi-model spatial feature fusion. Comput. Methods Programs Biomed. 208, 106221. Epub 2021 Jun 3. PMID: 34144251. 10.1016/j.cmpb.2021.106221 34144251

